# Opioid Use Disorder in Women and the Implications for Treatment

**DOI:** 10.1176/appi.prcp.20190051

**Published:** 2020-10-13

**Authors:** Celestina Barbosa-Leiker, Aimee N. C. Campbell, R. Kathryn McHugh, Constance Guille, Shelly F. Greenfield

**Affiliations:** College of Nursing, Washington State University, Spokane, (Barbosa-Leiker); Program of Excellence in Addictions Research, Washington State University, Spokane, (Barbosa-Leiker); Department of Psychiatry, Columbia University Irving Medical Center, New York State Psychiatric Institute, New York, (Campbell); Department of Psychiatry, McLean Hospital, Harvard Medical School, Boston, Massachusetts (McHugh, Greenfield); Department of Psychiatry and Behavioral Sciences and Obstetrics and Gynecology, Medical University of South Carolina, Charleston, (Guille).

## Abstract

**Objective:**

The opioid epidemic continues to evolve and impact all groups of people. Moreover, there are concerning trends among women. The aim of this article is to provide a review of opioid use disorder in women and the implications for treatment.

**Methods:**

A nonsystematic review of the literature as conducted to examine: (1) the epidemiology of opioid-related hospitalizations and deaths of women; (2) co-occurring pain, anxiety disorders, and trauma among women with opioid use disorder; (3) evidence for opioid agonist treatment of pregnant women with opioid use disorder; and (4) implications for treatment of women with opioid use disorder and next steps for research and practice.

**Results:**

The current opioid epidemic has produced important differences by sex and gender with increased rates of use and overdose deaths in women. Significant mental health concerns for women include co-occurring psychiatric disorders and suicide. Expanding medication treatment for perinatal opioid use disorder is crucial. While effective treatments exist for opioid use disorder, they are often not accessible, and a minority of patients are treated.

**Conclusions:**

The end to the opioid epidemic will require innovative multi-systemic solutions. There are significant practice gaps in preventing rising death rates among women by opioid overdose, treating co-occurring psychiatric disorders and pain, and treating perinatal women with opioid use disorder and their infants. Research on sex and gender differences, and the intersection with race/ethnicity and US region, is critically needed and should include treatment implementation studies to achieve wider access for women to effective prevention, early intervention, and treatment.

The opioid epidemic is evolving, intensifying, and impacting all groups of people. Moreover, there are concerning trends among women (1–4). The end to the opioid epidemic will require treatment solutions that consider women’s risks and needs as they relate to opioid use disorder and co-occurring conditions. It is crucial to identify practice gaps in preventing opioid overdose deaths among women, treating co-occurring psychiatric disorders and pain, and treating perinatal women with an opioid use disorder.

Therefore, a nonsystematic review of the literature was conducted to examine the overarching theme of treatment implications for women with an opioid use disorder. Specifically, we aimed to address the following interrelated factors: the epidemiology of opioid-related hospitalizations and deaths of women; co-occurring pain, anxiety disorders, and trauma among women with opioid use disorder; evidence for opioid agonist treatment of pregnant women with opioid use disorder; and implications for treatment of women with opioid use disorder and next steps for research and practice. This review was authored by members of the National Institute on Drug Abuse (NIDA) Clinical Trials Network (CTN) Gender Special Interest Group, a group comprised of investigators and providers from throughout the network who provide consultation on CTN study design, methods and analysis to support the NIH policy of inclusion of women (5). This review reflects ongoing research focused on sex and gender differences in substance use disorders. We use “sex” to refer to the biological differences between females and males and “gender” as a social construct enacted through roles and behaviors in a cultural context (6), noting research on the intersection of sex and gender is lacking and is needed to fully understand substance use disorder treatment implications.

## EPIDEMIOLOGY OF OPIOID USE DISORDER IN WOMEN

Although more men die from prescription opioid overdoses than women each year, since 1999 deaths from prescription opioid overdoses increased 642% among women compared with a 439% increase among men (7). The drug overdose death rate for women increased 260% from 1999 to 2017 for women 30–64 years of age and the average age at death for drug overdose deaths increased by 3 years for women within in this age group (2), demonstrating a critical need for prevention and treatment specifically among middle-aged women.

While opioid prescribing rates have leveled off and are now declining, heroin and fentanyl are now increasingly contributing to the opioid overdose crisis. Heroin deaths among women increased at more than twice the rate than among men from 1995 to 2015 (4). In addition, there has been a drastic increase in the rates of synthetic opioid-related deaths; these deaths increased 850% in women between 1999 and 2015 (3). Recent data focused on 30–64 year-old women from 1999 to 2017 show severe increases in overdose deaths involving synthetic opioids (1643%) and heroin (915%) (2).

In 2017, the age-adjusted death rate from drug overdoses involving opioids (per 100,000) was 6.1 for men and 4.2 for women (8). By United States region, age-adjusted death rates from drug overdoses involving opioids were highest for the Northeast (29.8), followed by the Midwest (25.4), South (20.7), and West (14.7). By race/ethnicity, age-adjusted death rates from drug overdoses involving opioids were highest for non-Hispanic White (27.5), followed by American Indian/Alaska Native (25.7), Black (20.6), Hispanic (10.6), and Asian/Pacific Islander (3.5).

Specific to prescription opioid overdoses (per 100,000) in 2017, age-adjusted death rates were 6.1 men and 4.2 for women, the same rates found for overdoses involving any opioid (8). However, different patterns emerge across overdoses involving prescription opioids when looking at United States region and by race/ethnicity. By United States region, age-adjusted death rates from drug overdoses involving prescription opioids were highest for the South (5.6), followed by the Midwest (5.5), Northeast (5.3), and West (4.1). By race/ethnicity, age-adjusted death rates from drug overdoses involving prescription opioids were highest for American Indian/Alaska Native (7.2), followed by non-Hispanic White (6.9), Black (3.5), Hispanic (2.2), and Asian/Pacific Islander (0.6).

While national surveillance data allows us to view patterns of rates, data reflecting the intersection of gender, United States region, and race/ethnicity (e.g., the rate of opioid overdose for Black women in the West) is largely missing, with few exceptions. Examining opioid overdose deaths by race/ethnicity and gender offers a closer look at disparities among vulnerable groups. Compared to the most common drug classifications, opioids were the most common contributor to overdose deaths for White women, White men, and Hispanic women from 2000 to 2015 (9). The largest recent increases in drug overdose death for Black and Hispanic women and men were due to heroin, and there were also increases for natural/semisynthetic and synthetic opioids.

When examining data on women by rurality and United States region, a study published in 2012 found that death rates from drug overdose in 2008 for women were more pronounced in rural areas in the South and Midwest, whereas prescription opioid rates were highest in Appalachia, along with counties in Southern and Western states (10). Opioid-related hospital stays in 2016 demonstrate regional, racial, age, and urbanization differences for women. Overall, White women had the highest rate of opioid-related hospital stays across all income levels, followed by Black women, but the difference between rate of stays between White and Black women were less pronounced in higher income quartiles (11). White and Black women had a similar rate of opioid-related stays in large metropolitan areas, yet in rural areas, Black women had a lower rate of opioid stays compared to White women. In western and north central United States, opioid-related hospital stays were higher among older (65 yearsþ) women, but in the northeastern United States, these rates were higher among younger (15–44 years) women. Co-occurring mental disorders were present with 56.5% of opioid-related hospital stays compared with only 26.5% of non-opioid-related stays for women, and White women had a somewhat higher percentage of co-occurring mental disorders (59.1%) compared with other racial/ethnic groups (range: 46.4%–52.0%).

The epidemiology of opioid use disorder in women points to an evolving epidemic that is impacting every demographic group across the nation, with severe consequences and significant rising rates of opioid use disorder and overdose among women across the United States. It appears that while White women and men may be at risk for overdose from any opioid, Black and Hispanic women and men may be at risk for overdose from heroin, and American Indian/Alaska Native women and men may be at risk for overdose due to prescription opioids. However, misclassification of Hispanic origin in surveillance data should lead to caution when interpreting data (8). Additionally, when considering United States region, it appears the Northeast has the highest overall opioid overdose rate and the South has the highest prescription overdose rate. Lastly, ethnic disparities in rural areas may be more pronounced with respect to opioid-related hospital stays. However, data broken down by sex/gender, race/ethnicity, and Unites States region, is the exception. We need national surveillance data on opioid overdoses that intersect sex/gender, race/ethnicity, and Unites States region to better target often scarce treatment resources.

Considerations for comorbidities, treatment of pregnant women, and “telescoping,” the accelerated progression from the initiation of substance use to the onset of dependence and first admission to treatment (12), highlight the complexities and distinctiveness of opioid use disorder among women and the critical need for tailored prevention and treatment. The next sections cover these crucial topics.

## OPIOID USE DISORDER TREATMENT CONSIDERATIONS FOR WOMEN

Studies have consistently indicated that treatment for opioid use disorder is effective in both men and women, without evidence of a sex difference in opioid use outcomes (13, 14). This suggests that front-line treatment for opioid use disorder can be used with confidence in both men and women. However, there are sex and gender differences in presenting characteristics (which may indicate different treatment needs) and in secondary outcomes that have implications for the effective treatment of opioid use disorder in men and women.

For example, in a comparative effectiveness trial of extended-release naltrexone (XR-naltrexone) versus buprenorphine for opioid treatment (i.e., *X:BOT*) for individuals (N=570) with opioid use disorder (13), baseline data demonstrated significant gender differences in demographic and clinical characteristics (15). This study showed that women with opioid use disorder compared to men with opioid use disorder were significantly younger, less likely to be employed, more likely to identify as bisexual, live with a sexual partner or with children alone, live with someone who has a drug problem, and depend on someone else for the majority of their support. Women’s duration of opioid use was shorter than men’s (M=11.2 years vs. M=13.1 years), despite no difference in the age of opioid use onset (M=21.3). Fewer women reported heroin as their primary opioid and women reported greater number of days of other opioid use in the past month. Women also reported fewer lifetime drug treatments. No differences were detected by gender for cigarette smoking, money spent on opioids per day, days of heroin use in past month, subjective reporting of opioid withdrawal discomfort, use of stimulants, benzodiazepines, and cannabis, preference for buprenorphine or XR-naltrexone, or likelihood of a previous opioid use disorder medication treatment. Significantly more women than men reported exchanging sex for drugs and sharing injection equipment; however, there was no difference in concern about human immunodeficiency virus (HIV) transmission. The data indicate that women are disproportionately affected by an array of psychosocial needs, fewer socioeconomic resources, and greater vulnerability for unsafe sex and drug practices, and exhibit similar opioid use disorder severity, yet have received less treatment relative to men.

Among the most consistently replicated sex and gender differences in substance use disorder research is that women are disproportionately affected by co-occurring psychiatric disorders (16). With opioid use disorders, co-occurring psychiatric disorders are associated with greater clinical severity and impairment, and when untreated, are associated with poorer functional outcomes (17). Women are more likely than men to be diagnosed with anxiety and depressive disorders both in the general population (18) and among people with opioid use disorder (14,19). For example, in a large clinical trial (N=653) of adults with prescription opioid use disorder, women were more than twice as likely as men to have a lifetime diagnosis of major depressive disorder (47.5% vs. 20.2%) or posttraumatic stress disorder (11.9% vs. 5.4%) (14). Similarly, in the *X-BOT* trial, women compared to men were significantly more likely to report a psychiatric history (including anxiety, bipolar, and depression disorders), history of suicidal behavior, physical and sexual abuse, as well as more days in the past month where their mental health was “not good” (15). Childhood trauma is also more common in women than men (20). Women in the general population are more likely to suffer from chronic pain (21); however, clinical trials of men and women with opioid use disorder have often not detected differences in the prevalence of chronic pain (14, 15).

In addition to their implications for severity and functioning, these disorders may also worsen opioid use disorder course. Women with opioid use disorder report more frequently using opioids to cope with negative effect or pain (14), and thus untreated co-occurring disorders may increase risk for ongoing opioid use. Furthermore, a major and growing concern for women with opioid use disorder and co-occurring disorders is benzodiazepine misuse. The concurrent use of benzodiazepines and opioids is a significant risk for overdose; approximately 30% of opioid overdose deaths also involve a benzodiazepine (22). Approximately 70%–75% of people with opioid use disorder misuse benzodiazepines at some point in their lifetime (23). In an analysis of National Survey on Drug Use and Health data, we found that women with opioid use disorder were 64% more likely to misuse benzodiazepines than men (23) and the CDC recently reported an increase of 830% in benzodiazepine overdose deaths among women from 1999 to 2017 (24).

Thus, women are more likely to have co-occurring disorders, more likely to use opioids to cope with these symptoms and are at a particularly heightened risk for misusing medications that are commonly used to treat co-occurring psychiatric disorders but also raise the risk of overdose. However, little is currently known about the optimal treatment for co-occurring psychiatric and pain-related disorders in people with opioid use disorder in general, or women specifically. Data on other substance use disorders suggests that integrated treatment (i.e., treatment that targets multiple co-occurring disorders together) is a particularly effective intervention for co-occurring substance use and psychiatric disorders (25, 26). Further research specifically on the treatment of opioid use disorder, including studies of psychiatric intervention in people receiving medication for opioid use disorder is much needed. Although much remains to be understood about the optimal treatments for co-occurring opioid use disorder and psychiatric disorders, several potential treatment targets warrant consideration.

First, many of the conditions that commonly co-occur with opioid use disorder in women (anxiety disorders, depressive disorders, and post traumatic stress disorder (PTSD)) share in common heightened distress intolerance, or the perceived inability to cope with distressing emotional (e.g., anxiety and frustration) and somatic (e.g., pain) symptoms. People with opioid use disorder report heightened distress intolerance compared to psychiatrically matched comparison groups (27), and distress intolerance distinguishes people with chronic pain who use their medication as prescribed from those who misuse their medication (28). Distress intolerance is hypothesized to heighten the motivation for behaviors that afford quick, proximal relief of distress, such as substance use. Recent data have suggested that among people with opioid use disorder, intolerance of anxiety is associated with benzodiazepine misuse *only in women* (29). Accordingly, distress intolerance may be an important transdiagnostic treatment target that is pertinent to both opioid use disorder and common co-occurring conditions. While gender disparities in co-occurring psychiatric disorders exist, both men and women with opioid use disorder are impacted by psychiatric comorbidity. When left untreated, such disorders and symptoms may result in continued impairment and distress that cannot be addressed by treating the opioid use disorder alone.

In addition to sex and gender differences in psychiatric comorbidity, data also underscore the economic vulnerability, high risk for infection and other complications of opioid use among women, and specific risk among sexual minority women (15). Gender-specific interventions for women with opioid use disorder should be considered, including integration of opioid use disorder care with treatment for co-occurring psychiatric disorders and trauma, risk reduction interventions which address relational dynamics, and interventions that address the unique needs of sexual minority women. Sex and gender differences in psychosocial needs, such as financial stress and childcare, should also be considered; women may face greater barriers to entering and remaining in care due to these types of factors. Treatments that can be applied to the multiple presenting conditions that often characterize this population (e.g., anxiety disorders, depression, chronic pain, etc.) may be particularly important for women (30, 31). Thus, comprehensive psychiatric assessment should be systematically included in opioid use disorder treatment across genders.

## TREATMENT OF PERINATAL OPIOID USE DISORDER

According to the Center for Disease Control and Prevention the number of pregnant women with opioid use disorder at labor and delivery increased by more than fourfold, increasing from 1.5 per 1000 delivery hospitalizations in 1999, to 6.5 per 1000 delivery hospitalizations in 2014 (32). Prenatal opioid use disorder is associated with considerable maternal, obstetric, fetal, and newborn morbidity and mortality. Women with a diagnosis of opioid use disorder at the time of delivery are 4.6 times more likely to die, 3.5 times more likely to have a cardiac arrest, and twice as likely to have intrauterine growth restriction, placental abruption, prematurity, blood transfusion, stillbirth, cesarean section, and preeclampsia or eclampsia, compared to women without a diagnosis of opioid use disorder even after controlling for a number of significant potentially confounding variables (33).

With this rise in non-medical use of opioids during pregnancy, there has been a sharp increase in Neonatal Opioid Withdrawal Syndrome (NOWS). NOWS, formerly known as neonatal abstinence syndrome can occur as a result of opioid use during pregnancy including licit and illicit opioid use or pharmacotherapy used to treat an opioid use disorder (e.g., buprenorphine and methadone). In the United States, the incidence of NOWS has increased substantially over the past decade. From 2004 to 2014 the incidence of NOWS increased from 1.5 per 1000 hospital births to 8.0 per 1000 hospital births (34). Some areas of the country have been particularly affected by the opioid epidemic including New England and Appalachia (35). In 2017, West Virginia had the highest rate of NOWS with approximately 5.12% of live births—a rate that is 10 times the national average—and an overall intrauterine substance exposure of 13.99%. The increase in NOWS has taken place among hospital births financed by both private and public insurance, but the increase in NOWS is even more pronounced in Medicaid recipients. The incidence of Medicaid-financed births increased from 2.8 per 1000 hospital birth in 2004 to 14.4 per 1000 hospital births in 2014 (34), which may reflect biases in screening for substance use among pregnant women depending on insurance status.

The standard of care for the treatment of perinatal opioid use disorder includes a comprehensive treatment program with prenatal care, addiction treatment services, pharmacotherapy, and interventions to address comorbid mental health, trauma and social needs (i.e., housing instability and food scarcity) (36). Pharmacotherapy for the treatment of perinatal opioid use disorder includes buprenorphine or methadone. Methadone has been the recommended standard of care since the early 1990s (37) and buprenorphine has been considered another appropriate treatment options since 2010 (38).

A Cochran systematic review comparing the efficacy of methadone verses buprenorphine for the treatment of perinatal opioid use disorder suggests that one medication is not superior to the other (39). Treatment retention rates appeared to be slightly higher in women receiving methadone compared to buprenorphine but the body of evidence is too small to draw definitive conclusions (39). Other systematic reviews and meta-analyses as well as a randomized controlled trial comparing short-term obstetric and newborn outcomes among infants exposed to buprenorphine or methadone for the treatment of prenatal opioid use disorder generally support buprenorphine as having a more favorable risk profile (40). For example, newborns exposed to buprenorphine, compared to methadone, have a lower risk for preterm birth and have a greater birth weight, and larger head circumference (40). In addition, prenatal buprenorphine treatment for opioid use disorder is associated with a lower risk for treatment of NOWS, requiring less medication and a shorter hospital stay, compared to newborns with in-utero exposure to methadone (38). While there may be subtle advantages and disadvantages to the use of methadone or buprenorphine during pregnancy, much of the decision to choose one medication over the other is due to patient preference, feasibility, availability that can vary widely across states and communities, and prior treatment response.

Several professional organizations focusing on women’s health and addiction including the American College of Obstetrics and Gynecology, the American Academy of Addiction Medicine, the Substance Abuse and Mental Health Services Administration and the World Health Organization all recommend that pregnant women with opioid use disorder receive pharmacotherapy (methadone or buprenorphine) and recommend against medication-assisted withdrawal, or opioid taper or detoxification (36). The rationale for this recommendation is based on studies demonstrating a very high risk for relapse to drug use with medication-assisted withdrawal. Although there are obstetric and newborn risks associated with methadone or buprenorphine, relapse to drug use places women at high risk for infectious diseases, exposures to violence, legal consequences and poor obstetric outcomes. As such, experts conclude that the risks of relapse to opioid use far outweigh the risks of medications used to treat opioid use disorder (36).

Despite the recommendation against medication-assisted withdrawal, some women may want to decrease their dose of methadone or buprenorphine or discontinue this medication with the hope to mitigate the risk of NOWS. It is critically important that clinicians present balanced information about the risks and benefits of medication assisted withdrawal and risks of methadone or buprenorphine so that women can make informed treatment choices. A shared decision-making tool is available to assist patients and providers in this treatment choice (41). As with all patients with opioid use disorder, pregnant women should also be prescribed naloxone and partners and family member should be educated on the administration of this drug as the risks associated with perinatal withdrawal with the administration of naloxone is appropriate in the context of a life-threating overdose.

The characteristics of optimal care for pregnant women with opioid use disorder during pregnancy and delivery, as well as in the critical 12–24 months following birth, requires additional research. For example, a new study will consider maternal and neonatal outcomes during pregnancy and in the critical 12 months following birth. This new multisite randomized trial titled “Medication treatment for Opioid Use Disorder in Expectant Mothers: a pragmatic randomized trial comparing extended-release and daily buprenorphine formulations” (ClinicalTrials.gov Identifier: NCT03918850) is beginning implementation in 2020 and will ultimately provide critical data on both maternal and newborn outcomes in the year following birth (42).

## WHAT WE KNOW AND WHERE WE NEED TO GO

Opioid use disorder and opioid related deaths in both men and women continue to rise (43) and despite effective treatments, only about 20% of individuals are treated each year (44). One of the significant challenges in closing this treatment gap is that the epidemic is complex and changing demographically and geographically. While some data exist that allow us to view data by gender, race/ethnicity, and United States region, data reflecting the intersection of these categories is largely missing. Without data reflecting additional information about women and the intersection of race/ethnicity, gender and region, we continue to have limited knowledge of the most effective approaches to screening and treatment for diverse subgroups of women with an opioid use disorder in different regions across the nation. In order to reduce disparities for women of color and sexual minority women, greater inclusion of these groups in clinical trials and other research is much needed. It will be important to reduce barriers to health care access for these groups and to provide culturally appropriate and acceptable screening and treatment. Stigma, racism, and discrimination remain obstacles for health equity including Black, Indigenous, and other women of color who have an opioid use disorder.

There is rising prevalence of opioid use disorder among women through the lifespan including women of reproductive age (45). It has become increasingly clear that widening access to effective treatment for the entire population affected by opioid use disorder, including women and their families, will require multifaceted innovation, increased funding, effective approaches to treatment implementation, and ongoing research to advance additional solutions.

One of the most critical differences between the effects of this epidemic on women compared with those of other substance use disorders is the unprecedented urgent and rising death rate of women from opioids that has been accelerating even more steeply since 2016 (43). This increase may in part reflect an overlapping rise in completed suicides among women that involve opioid poisonings that may be misclassified as “accidents” (46). Opioid use disorder itself is associated with risk of completed suicide (47, 48). While completed suicides are still more prevalent in men than women, there has been a sharp increase in opioid poisoning deaths among women that may represent an increase not only in “accidental” overdoses but in completed suicide. Suicide risk may also be heightened in women with opioid use disorder due to the prevalence of chronic pain, depression, anxiety, and traumatic stress (47).

The opioid epidemic has highlighted critical health disparities for pregnant women with opioid use disorder that often have little or no access to comprehensive treatment services that can provide methadone or buprenorphine treatment to increase the likelihood of healthy pregnancies and deliveries. Comprehensive treatment programs providing agonist treatment for opioid use disorder in pregnancy increases the likelihood that the delivery will take place in the same hospital where prenatal care is delivered. It also gives opportunity for advanced planning for care of the mother and the newborn at birth. While women can receive Medicaid coverage for services during pregnancy, they often lose insurance after the baby is born, further complicating their ability to continue their engagement with medications and other treatments for their opioid use disorder. Ideally, given that the time following birth is high risk for both mother and infant, the health system should provide comprehensive treatment for mother and infant (to include evidence-based addiction treatment, early intervention for the baby, parenting skills and family services, and stable housing) not only in the immediate post-partum period, but for 24 months following delivery—a critical time period for the mother’s risk for opioid use disorder relapse and for infant development. While we currently lack prospective studies of infants born with NOWS, given the social and economic circumstances of many of these infants, as well as other potential prenatal exposures (i.e., tobacco smoking, other substances, and lack of prenatal care), these infants may continue to be at neurodevelopmental risk requiring additional interventions.

While pregnancy itself is often a barrier for substance use disorder treatment, women with opioid use disorder share many other clinical characteristics as well as well-known barriers to treatment that face women with other substance use disorders. Women with opioid use disorder are less likely to receive treatment yet experience a telescoping course of illness such that they experience more severe medical and psychosocial consequences of their opioid use disorder often with fewer years of use. In addition to co-occurring psychiatric disorders, other gender-specific barriers to obtaining opioid use disorder treatment for women that are consistent with other substance use disorders include trauma histories, financial vulnerability, lack of transportation, a partner who is also using substances, lower levels of social support, and higher levels of stigma and discrimination including punitive legal actions in many states for pregnant women with substance use disorders. Pregnant and parenting status can also present women with an additional barrier to obtaining treatment due to lack of childcare (30).

In addition, women are more likely to be prescribed prescription opioids, given higher doses, use them for longer time periods, and become dependent more rapidly (3). Women who develop prescription opioid use disorder are more likely to have chronic pain, other co-occurring psychiatric disorders, and decreased functional status compared with men. They are more likely to obtain prescription opioids from family and friends while men are more likely to purchase them (49). In 2010, significantly fewer women than men had obtained any treatment for an opioid use disorder than men (49); however, one 2017 study demonstrated that this gap may be narrowing especially in inpatient treatment for an opioid use disorder (50).

The largest randomized controlled trial of prescription opioid use disorder treatment is the Prescription Opioid Addiction Treatment Study (51). Individuals (N=653; 40% women) with prescription opioid use disorder were randomized to two buprenorphine taper schedules. No overall gender differences in opioid dependence severity at baseline or main treatment outcome were observed. However, women had greater functional impairment, psychiatric severity, and were more likely to use prescription opioids to cope with negative effect and pain, while men had more opioid craving and significant alcohol misuse than women (14, 51).

There are specific implications for treatment of women with opioid use disorder. It is especially important for women with opioid use disorder to be able to obtain comprehensive treatment of not only the opioid use disorder but also co-occurring depression, anxiety, and PTSD to increase the likelihood of optimal treatment for both the opioid use disorder and the other psychiatric disorders. There is a need to target suicide prevention efforts as well through comprehensive treatment of all these disorders. Gender-specific treatment for women with opioid use disorder must also address the central role that relationships with children, intimate partners, and others play in women’s addiction and recovery; as well as the physical vulnerability to medical illness, and the needs of pregnant and post-partum women.

Gender-specific treatment may include women-only treatment programs but often women seek opioid use disorder treatment in mixed-gender settings. Gender-responsive treatment in mixed-gender settings can include components of care that focus on clinical characteristics that are of special significance to women with substance use disorders. For example, The Women’s Recovery Group (WRG) was developed in Stage I and Stage II therapy development trials funded by NIDA (30, 31). It is a manualized, relapse-prevention group therapy with structured sessions, and women-focused content. The WRG is an empirically supported, gender-responsive component of care that can be disseminated into routine clinical practice and is designed for women who are heterogeneous with respect to the substances used; co-occurring other psychiatric disorders; partner status and sexual orientation; pregnancy or parenting status; trauma histories; among other clinical and demographic factors that are of significance to women’s addiction and recovery (52). Future studies may determine whether the addition of the WRG to medication treatment for opioid use disorder could enhance engagement, treatment, and retention with medication treatment for opioid use disorder.

Finally, there are important areas of research needed to investigate the most effective strategies for closing gaps for prevention, early intervention, and treatment for women with opioid use disorder. For example, we do not know the best models of expanding access through integration of treatment services within the health system for women (and their partners, children, and families) including medical services in obstetrics/gynecology, primary care, pediatrics, emergency medicine, psychiatry, and specialty addiction services. Additional studies could determine whether gender-specific components of care can enhance treatment outcomes for women with opioid use disorder such as induction and maintenance on medication treatment for opioid use disorder. Models of care for pregnant women with opioid use disorder would benefit from focus on the integrated treatment of pregnant and post-partum women and their children including relapse prevention during and after pregnancy as well as follow-up for mothers and their infants 24 months post-partum. Additional areas of investigation that would be important include the best models of implementing comprehensive treatment of opioid use disorder with treatment of co-occurring PTSD, depression, and anxiety as well as targeted suicide prevention approaches. Critical studies could also focus on effective interventions to decrease drug taking and sex risk behaviors in women with opioid use disorder examining both opioid use disorder treatment outcomes as well as decrease in transmission of infectious diseases such as hepatitis C and HIV. The experience with the COVID-19 pandemic has also demonstrated that telehealth and telepsychiatry can be effective approaches to extend treatment to individuals who do not otherwise have opportunity to receive in-person evaluation and treatment. Telehealth and digital interventions are likely to be critical components of care to expand treatment and reduce disparities for women with opioid use disorders across race/ethnicity and geographic region. More research is needed, however, to understand for whom telehealth may not be as promising (e.g., those with unstable housing or lower economic resources who lack confidential spaces, appropriate technology, or data plans). Finally, research that provides only a binary assessment of gender/sex differences of main outcomes of large clinical trials of pharmacotherapies is necessary but not sufficient in examining sex and gender differences in substance use disorders including those for an opioid use disorder. Gender-specific data on clinical characteristics such as co-occurring disorders, trauma histories, sex risk behaviors, social supports, economic circumstances, transportation, partners, and dependent children, is critical to reduce disparities and design effective treatment services for retention, relapse prevention, and well-being.

## SUMMARY

Opioid use, opioid use disorder, and opioid-related deaths continue to rise, and there are several concerning trends among women such as a larger increase in overdose deaths relative to men. While effective treatments exist, they are often not accessible, and a minority of patients are treated. Further, women face complex challenges with regard to mental health, pain, pregnancy and parenting, and economic vulnerability that require unique and innovative interventions. The end to this epidemic will thus require multi-component and complex solutions as well as changes that are both attitudinal and systemic, and research on sex and gender differences is needed to move forward toward this goal. Such research will need to include treatment implementation studies to achieve wider access to and acceptability of effective prevention, early intervention, and treatment.

## HIGHLIGHTS

- Significant mental health concerns for women include co-occurring psychiatric disorders, pain, and suicide.
- Expanding medication treatment for perinatal opioid use disorder is crucial.
- Research on sex and gender differences, and the intersection with race/ethnicity and US region, is critically needed.
